# Influence of heating rate and soak time on microwave sintered hydroxyapatite and β-tricalcium phosphate ceramics for bone applications

**DOI:** 10.1038/s41598-025-30915-4

**Published:** 2025-12-11

**Authors:** Bhupesh Sarode, Abhaykumar Kuthe, Ankush D. Bhishnurkar, Ashutosh D. Bagde

**Affiliations:** 1 Department of Mechanical Engineering, Visvesvaraya National Institute of Technology (VNIT), Nagpur, 440 010 Maharashtra India; 2 Biomedical Engineering, Faculty of Engineering and Technology, Datta Meghe Institute of Higher Education and Research, Wardha (M.S.), 442004 India; 3 Jawaharlal Nehru Medical College, Datta Meghe Institute of Higher Education and Research, Wardha (M.S.), 442004 India

**Keywords:** Ceramic, HA-TCP, Heating rate, Microwave, Sintering, Engineering, Materials science

## Abstract

Calcium phosphate-based bio-ceramics, particularly hydroxyapatite (HA) and β-tricalcium phosphate (β-TCP), have gained prominence in biomedical engineering due to their biocompatibility and resemblance to natural bone mineral. However, the non-degradability of HA and the rapid resorption of β-TCP pose challenges for bone scaffold applications. Biphasic calcium phosphate (BCP) composites, combining HA and β-TCP, offer a promising solution by achieving a controlled degradation profile. In this study, HA-TCP ceramics were fabricated using a novel microwave sintering technique to investigate the influence of ramp temperature rate and soak time on the microstructural and mechanical properties of sintered pellets. A ceramic composite with an 80:20 weight ratio of TCP and HA was prepared, and a polyvinyl alcohol (PVA) binder was used to optimize pellet formation. The sintering process was conducted at 1200 °C under varying heating rates (15 °C/min, 25 °C/min, and 35 °C/min) and soak times (30, 45, and 60 min). Results revealed that a ramp temperature rate of 35 °C/min with a soak time of 45 min achieved optimal outcomes, including reduced porosity (26.158%) and increased compressive strength (39.15 MPa). Higher heating for a longer time leads to phase change from β-TCP to α-TCP. Additionally, prolonged soak time during slow cooling resulted in phase transformations from α-TCP to β-TCP, which impacted the mechanical properties. Microwave sintering demonstrated significant advantages, including reduced processing time, energy efficiency, and enhanced densification. This study establishes optimized parameters for the fabrication of HA-TCP ceramics with tailored porosity and mechanical properties, providing a foundation for the development of advanced bone scaffolds in biomedical engineering.

## Introduction

Calcium phosphate (CaP)-based bioceramics have long been recognized as promising candidates in medical engineering due to their remarkable compositional similarity to the mineral phase of natural bone^1,2^. Their exceptional biocompatibility has been consistently demonstrated in preclinical and clinical studies, positioning them as highly attractive materials for diverse biomedical applications, particularly in bone repair and regeneration^3–5^. In recent years, advanced CaP ceramics, such as hydroxyapatite (HA), α-tricalcium phosphate (α-TCP), β-tricalcium phosphate (β-TCP), and biphasic calcium phosphate (BCP), have been widely investigated in clinical trials as bone graft substitutes and scaffold materials^6,7^. Among these, biphasic HA–TCP systems have drawn particular attention because they integrate the stability of HA with the bioresorbability of TCP, providing a tunable degradation profile and superior osteoconductivity^8–11^.

Hydroxyapatite is chemically analogous to bone mineral and exhibits intrinsic bioactivity, which explains its broad use in orthopedic and dental applications. However, HA is essentially non-degradable under physiological conditions, limiting its suitability for applications requiring scaffold resorption and replacement by newly formed bone. In contrast, β-TCP is biodegradable and supports rapid remodeling but often degrades too quickly, compromising long-term mechanical stability^4,5,12^. BCP composites, comprising an optimized ratio of HA and β-TCP, have thus emerged as a rational strategy to achieve controlled resorption and sustained osteointegration^13^. Despite these advantages, CaP ceramics are inherently brittle and exhibit poor mechanical strength, which restricts their direct application in load-bearing environments^2^. Consequently, significant research has focused on optimizing both composition and processing routes to enhance their structural and biological performance.

Various processing methods have been employed to fabricate porous CaP scaffolds, including particle packing, injection molding^14^, foaming^15^, and the use of pore-forming agents^16^. Densification of CaP ceramics is typically achieved by conventional sintering^17,18^, spark plasma sintering^19–21^, freeze casting^22^, additive manufacturing^23^ and more recently, microwave sintering (MWS)^10,11,24^. Overcharging sintering of HA-TCP ceramics is essential to achieve complete densification, eliminate residual porosity, and enhance mechanical strength required for load-bearing biomedical applications. It promotes grain growth and phase transformations that improve the bonding between particles, resulting in a denser, mechanically robust scaffold with controlled bioresorbability^25,26^. Among these techniques, MWS has garnered considerable interest as an advanced consolidation strategy due to its unique attributes^27^, including volumetric heating, reduced sintering cycles, lower energy consumption, and enhanced densification efficiency^27^. During MWS, organic binders and volatile components in the green compact evaporate at intermediate temperatures, producing a porous ceramic framework^28,29^. Subsequent high-temperature sintering consolidates particles, enhances mechanical strength, and refines the crystalline microstructure, resulting in improved reliability of the scaffold^30–35^. Additionally, as a pressure-less process, MWS offers scalability and the potential for fabricating architected, near-net-shape structures, making it highly attractive for biomedical applications.

For HA–TCP specifically, MWS has demonstrated significant benefits over conventional sintering routes. Calcium phosphate ceramics rapidly heat under microwave irradiation because the phosphate ions and ionic species strongly interact with microwave energy, enabling efficient dielectric heating through ionic conduction and dipolar polarization. This leads to fast, uniform volumetric heating, resulting in significantly higher sintering temperatures and accelerated densification compared to conventional methods^36^. Rapid microwave heating suppresses grain coarsening, minimizes HA decomposition into TCP and CaO, and controls the β to α-TCP transformation, thereby maintaining phase stability while reducing cycle times from several hours to mere minutes^37,38^. These microstructural refinements result in nanocrystalline surfaces with higher specific surface area, which enhance protein adsorption, osteogenic marker expression, and in vivo bone regeneration. Sintered HA-TCP ceramics exhibit excellent biocompatibility, osteoconductivity, and promote osteogenic differentiation and bone regeneration in vitro and in vivo, supporting the biological relevance of our materials^39,40^. For instance, Xiangfeng Li^37^ reported that HA/β-TCP (2:8) granules sintered by microwave heating at 1050 °C for only 5 min exhibited superior osteoinductivity and spinal fusion efficacy compared with conventionally sintered counterparts. Such findings underscore the translational potential of MWS-fabricated BCP in orthopedic applications.

Recent innovations in MWS further extend its capabilities. Hybrid and resistive-coupled microwave systems have been introduced to improve coupling efficiency with low-loss CaP green bodies, enabling near-net-shape densification at lower apparent furnace temperatures^41^. Dual-frequency microwave sintering has been successfully employed to incorporate antibacterial agents homogeneously within HA scaffolds^42^, providing pathways toward multifunctional HA–TCP systems. Furthermore, insights from β-TCP reaction-sintering^43^ are informing the design of optimized microwave schedules to preserve desirable HA/TCP phase balance. Despite these promising developments, challenges such as field non-uniformity, thermal gradients in complex geometries, and reproducibility issues associated with dielectric variations remain barriers to scaling up. Addressing these through advanced furnace designs, multi-physics modeling, and real-time process monitoring is critical for industrial translation.

Overall, the convergence of compositional tailoring, innovative sintering strategies, and process control highlights microwave sintering as a compelling route for next-generation HA–TCP bioceramics. By combining superior manufacturability with controlled bioresorption and enhanced osteogenic response, MWS-fabricated BCP scaffolds present a strong opportunity for high-performance orthopedic and dental applications^38,44^.

In this study, the investigation offers a basis for understanding the influence of ramp temperature rate and soak time for microwave sintering of HA-TCP ceramic-based pellets, which is critical for their application in making bone scaffolds in biomedical engineering. The article elaborates on the results obtained from a pellet formed with 20% binder and a 3% PVA concentration. Microwave sintering at 1200 °C, with three different ramp temperature rates and three different soak times, is investigated. Microstructural and mechanical characterization using scanning electron microscopy (SEM), X-ray diffraction (XRD), and compressive strength measurement was performed to investigate the post-sintering behavior of the pellet.

## Materials and methods

The 80:20 weight ratio of β-TCP to HA was selected based on earlier studies^45^ reporting that this composition provides an optimal balance between bioactivity and resorption rate in biphasic calcium phosphate (BCP) ceramics. This specific ratio was selected to leverage the complementary properties of both ceramics, where TCP contributes bio-restorability, and HA enhances bioactivity and structural stability.

A binder is a temporary additive that holds TCP and HA in powdered form together. It decomposes completely at high temperatures during processing without altering the properties of the final material. Polyvinyl alcohol (PVA), a water-soluble polymer widely employed as a binder in ceramic processing, was used to prepare a paste with the ceramic powders. PVA (341584), having Mw 89,000–98,000 and 99+% hydrolyzed, is used in this study to make a binder. To investigate the effect of binder concentration on paste characteristics and pellet formation, seven distinct PVA solutions were prepared. These solutions were prepared by dissolving PVA in distilled water at concentrations ranging from 1% to 7% by weight, ensuring a comprehensive evaluation of binder effects. The PVA was dissolved using a magnetic stirrer at 50 °C at 500 rpm for 6 h to get a uniform binder solution.

**Fig. 1:**
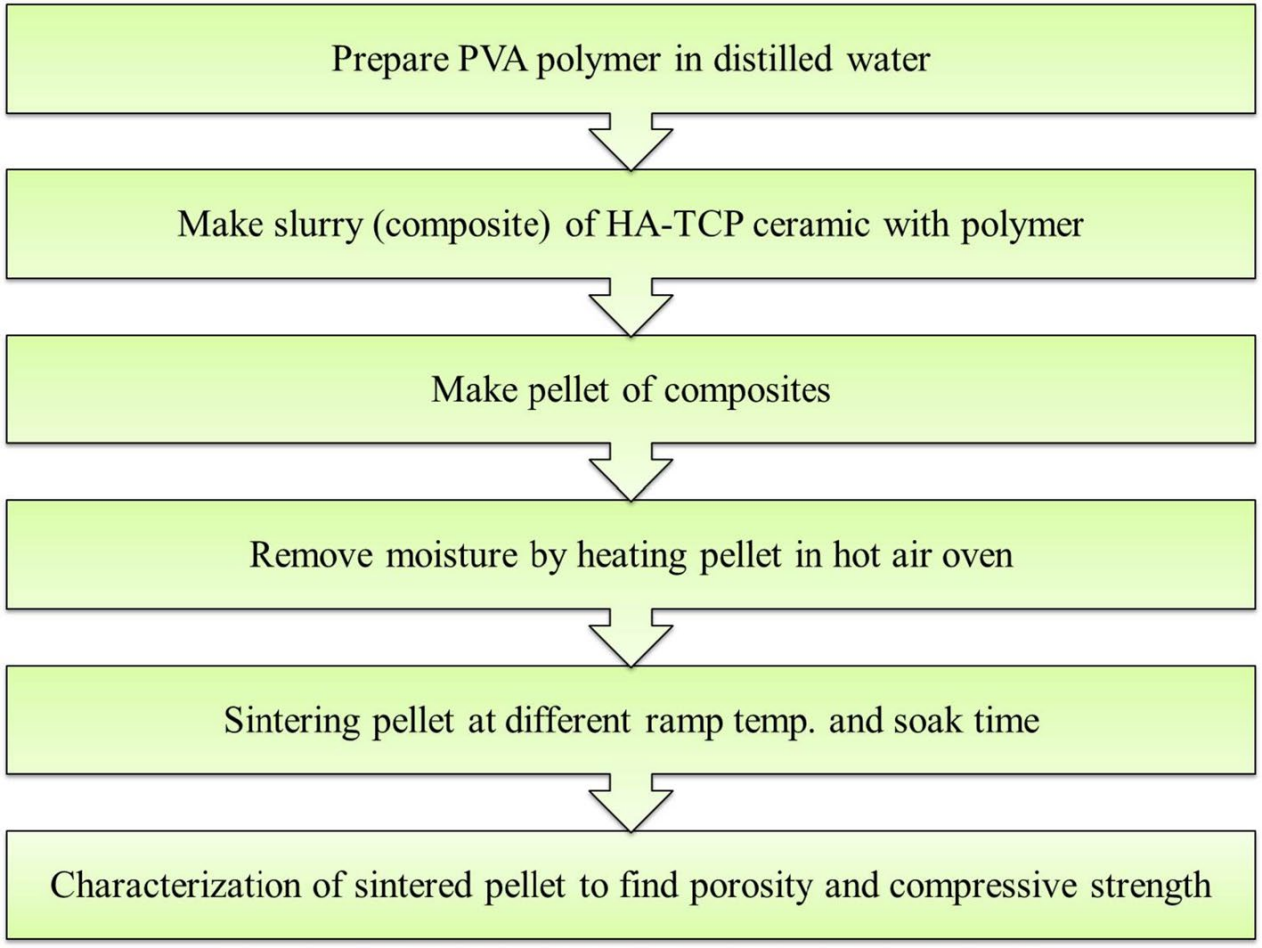
Pellet making process.

The ceramic powder paste was prepared by mixing the ceramic composite (TCP-HA) with each PVA solution at varying binder content levels of 10%, 20%, and 30% by weight. The paste was homogenized using an electric stirrer to ensure uniform distribution of the binder within the ceramic matrix. The prepared slurries were then used to fabricate cylindrical-shaped pellets of 10 mm diameter under controlled conditions using a hydraulic press with a range of 0.5 to 4 bar pressure. Pellets were dried in hot air oven to remove moisture and sintered at 1200 °C in a microwave applicator at 2.45 GHz. Figure 1 shows the complete procedure of pellet making, sintering and characterization, utilized for this study. Compressive testing of pellets was performed using a universal testing machine with a capacity of 10 kN, applying a controlled strain rate of 1.5 × 10^− 3^s^− 1^ in accordance with ASTM testing protocols.

All combinations were prepared and assessed in triplicate to ensure the feasibility and consistency of pellet formation. The resulting pellets underwent a qualitative visual inspection to assess the defect-free formation. Defects such as surface cracks, delamination, or uneven surfaces were taken into consideration during the evaluation process. The inspection results are summarized in Table 1, where “Y” indicates defect-free pellet formation, and “N” denotes the presence of defects. This visual inspection provides preliminary insights into the suitability of different binder concentrations for achieving defect-free pellets.

**Table 1:** Pellet formation with different forms of binder solution and PVA concentration.

Binder	PVA concentration (%)						
(%)	1	2	3	4	5	6	7
10	N	N	Y	Y	Y	Y	Y
20	Y	Y	Y	Y	N	N	N
30	N	N	N	N	N	N	N

### Experimental procedure

Paste of HA-TCP ceramic and PVA binder solution is formulated in a beaker using electric stirrer. Twenty-one different combinations, as shown in Table 1, were investigated to assess the paste’s ability to be drawn into a pellet. Excessive viscosity resulting from inadequate binder addition or excessive fluidity due to a surplus amount of binder addition leads to failure in pellet formation. A total of 12 combinations, including 2 due to high viscosity and 10 due to high fluidity, fail to produce good pellets out of 21 combinations.

**Fig. 2:**
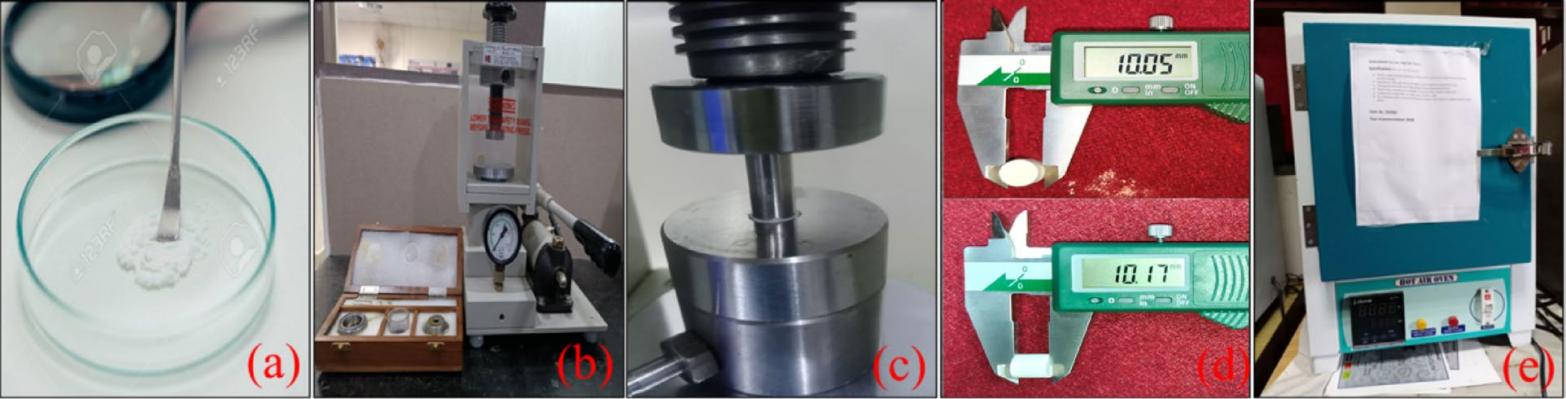
(**a**) Paste preparation, (**b**, **c**) pellet making machine, (**d**) pellet, (**e**) hot air oven.

A paste was poured into the hydraulic press pellet-making machine, and a pressure of 3 bar was applied to facilitate the formation of intermolecular bonding. The setup was kept at an ideal condition for 2 min, which allows the paste to transform into a pellet. The pellet was removed from the pellet-making machine and placed in a hot air oven for 60 min at 120 °C to remove its moisture content. Figure 2 shows the pellet-making process from the preparation of the paste to the removal of moisture in a hot air oven. The pellet was sintered in a multi-mode microwave applicator under ambient conditions, operating at a frequency of 2.45 GHz. An automated sintering program was executed, initially heating the pellets to 350 °C at a power of 0.5 kW. This was followed by a ramp to the target temperature of 1200 °C for a soak time duration of 30 min with ramp rates of 15 °C/min, 25 °C/min, and 35 °C/min, depending on the experimental condition. SiC susceptors were used to enhance uniform microwave absorption and heating efficiency. These parameters provide controlled and reproducible sintering conditions suitable for densification and microstructural optimization of HA-TCP ceramics.

The porosity of the sintered pellet was measured by porosity weighing kit using Archimedes’ principle with distilled water at room temperature. The pellet specimen was tested for compression strength using an axial load of 10 kN. The phase composition of the sample before and after sintering was analyzed via X-ray diffraction (XRD) (Rigaku Corporation, Japan). Gold particle spurting over the pellet was carried for analyzing the surface texture using Scanning Electron Microscopy (SEM).

## Results and discussion

All experiments were conducted in triplicate, and each characterization was systematically evaluated to confirm the reproducibility of the results.

### Effect of ramp temperature rate

**Fig. 3:**
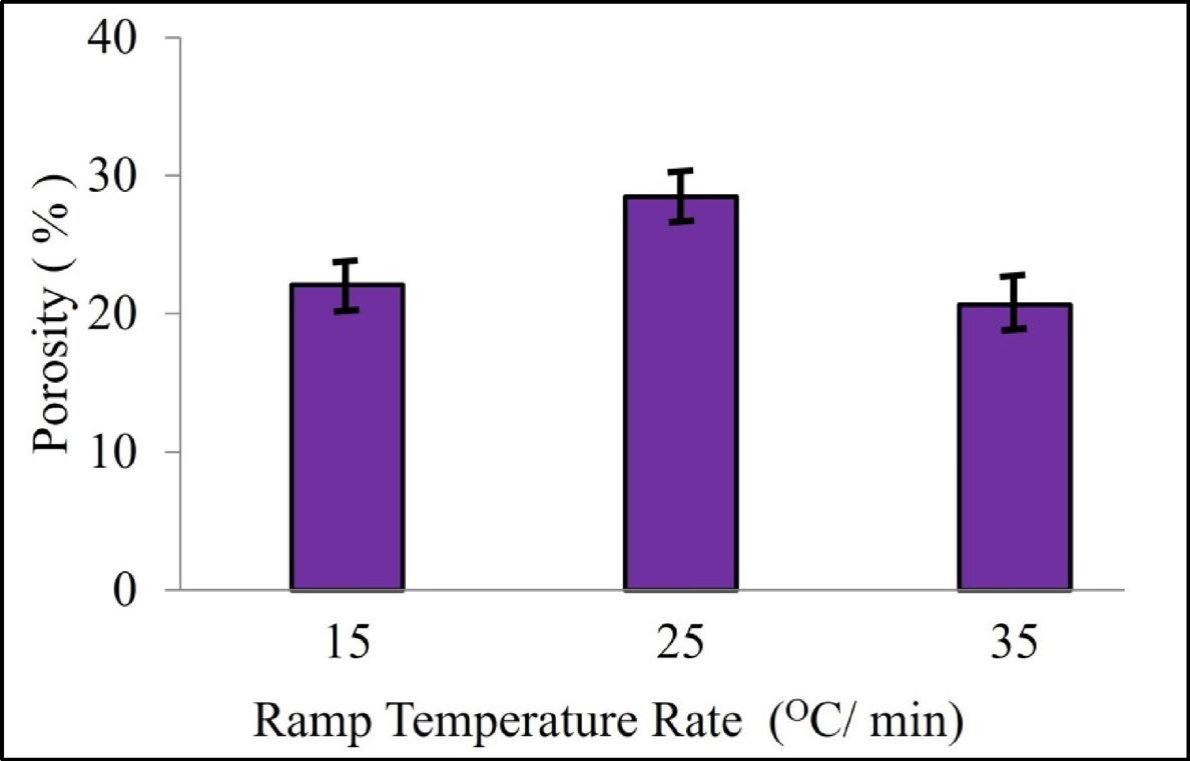
Porosity in sintered pellet with respect to change in ramp temperature rate.

**Fig. 4:**
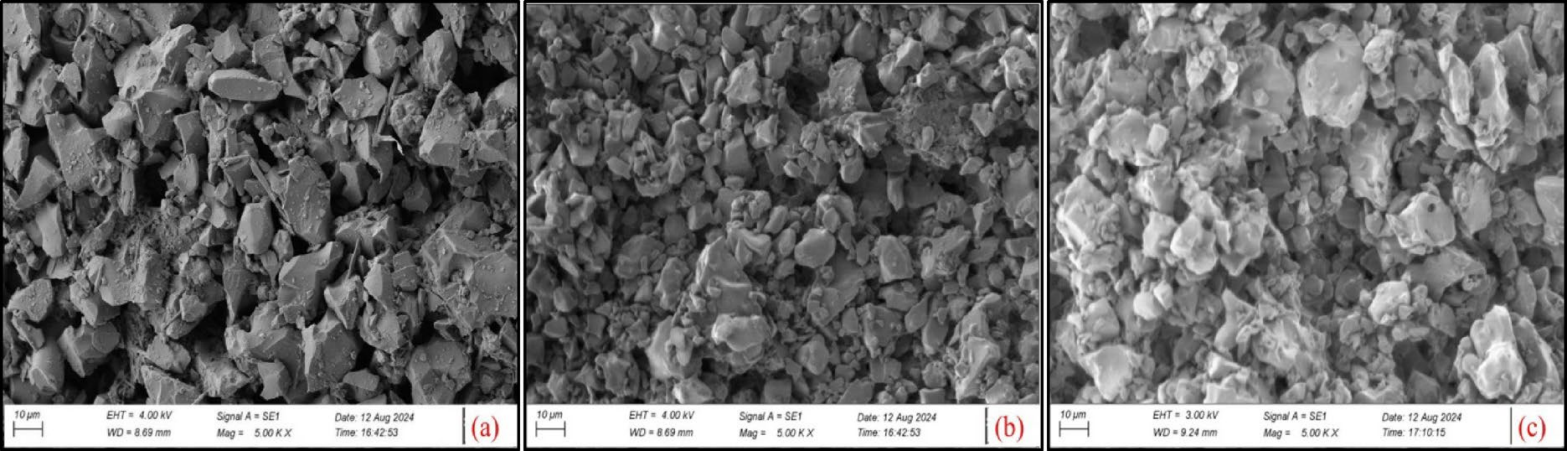
SEM of sintered pellet at ramp temperature rate of (**a**) 15 °C/min, (**b**) 25 °C/min, (**c**) 35 °C/min.

Results obtained from a pellet formed with 20% binder having 3% PVA concentration is discussed in this study. The porosity increases first and then decreases drastically with the increase in ramp temperature rate during sintering as shown in Fig. 3. The minimum relative density recorded was 71.55%, and the maximum relative density recorded was 79.342% for the ramp temperature rate of 25 °C/min and 35 °C/min, respectively. The High state of porosity is obtained at a ramp temperature rate of 25 °C/min, as lower temperature fails to provide complete sintering within ceramic pellet. Figure 4 shows the SEM micrographs of sintered pellets. The result indicates that a compact structure has been observed at a ramp temperature rate of 25 °C/min, and the porosity of the sample decreases as the ramp temperature rate increases. The results show that the higher liquid phase further shrinks and produces intermolecular bonding in the pellet material, making the sintered ceramic pellet denser. Similar results have been reported by Omayra Beatriz Ferreiro Balbuena^46^. The temperature of the SiC block remains higher than that of the HA-TCP ceramic during microwave sintering, as SiC has strong microwave absorptivity. Hence, it creates a high temperature zone at SiC and pellet junction area and promotes denser sintering of pellet.

**Fig. 5:**
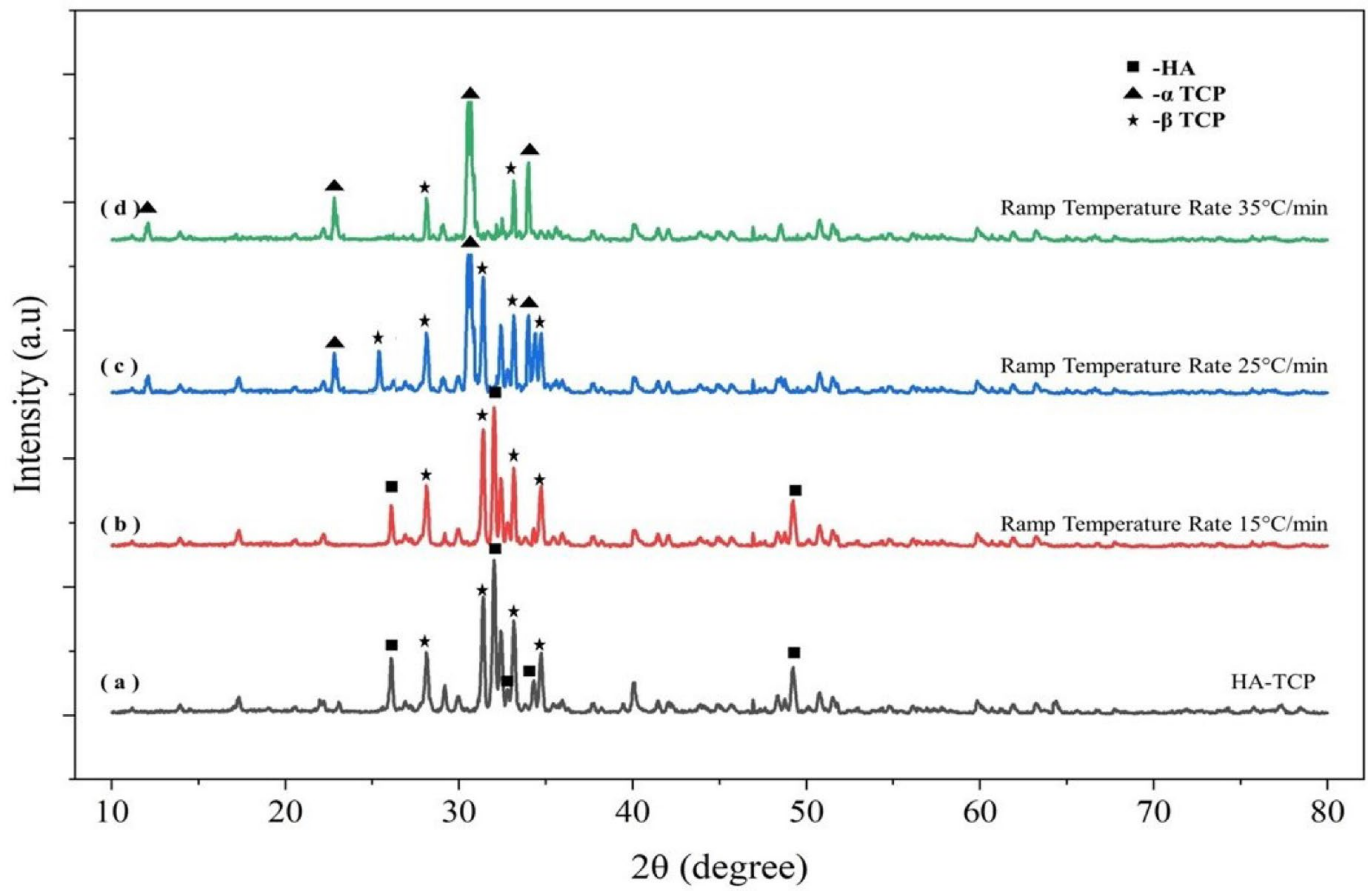
XRD of HA-TCP pellet and sintered pellet at different ramp temperature rates.

For an unpolished surface, it is observed in Fig. 4a that there is a presence of irregular-shaped powder particles, which are observed after sintering at the ramp temperature rate of 25 °C/min. Figure 5 shows the XRD results of HA-TCP ceramic mixture and sintered HA-TCP pellet. Figure 5b shows that the β-TCP phase (JCPDS No. 09-169) was observed in the sintered sample for the ramp temperature rate of 25 °C/min. This indicates the absence of liquidized areas, which enhances densification and transforms β-TCP to α-TCP (ICSD #29–0359) at a low ramp temperature rate. Figure 5c shows that most of the β-TCP is converted into α-TCP at a relatively higher ramp temperature rate, and there is a small amount of β-TCP in the sintered pellet, as shown in Fig. 5d.

**Fig. 6:**
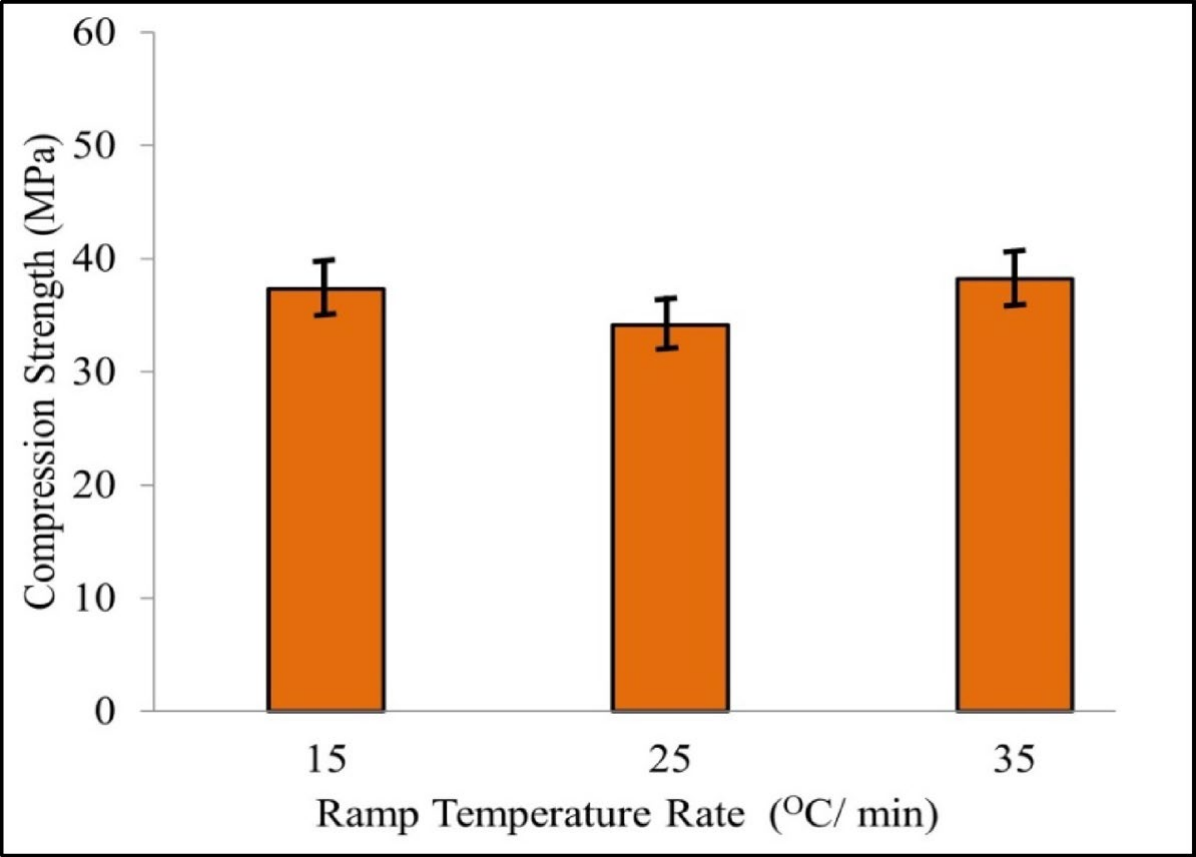
Compression strength of pellet for different ramp temperature rate.

As shown in Fig. 6, the compression strength reduces first and then increases as the ramp temperature rate of sintering increases. The trend in change for compression strength and porosity is the exact opposite. It was observed that denser region in the sintered HA-TCP pellet improves the mechanical properties. When the ramp temperature rate is 25 °C/min, the compression strength value is 34.157 MPa, and it reaches 38.21 MPa for a ramp temperature rate of 35 °C/min. As can be observed from Fig. 4b, the interlocking microstructure formed by α-TCP grains can greatly enhance the mechanical properties of the sintered pellet.

### Influence of soak time

**Fig. 7:**
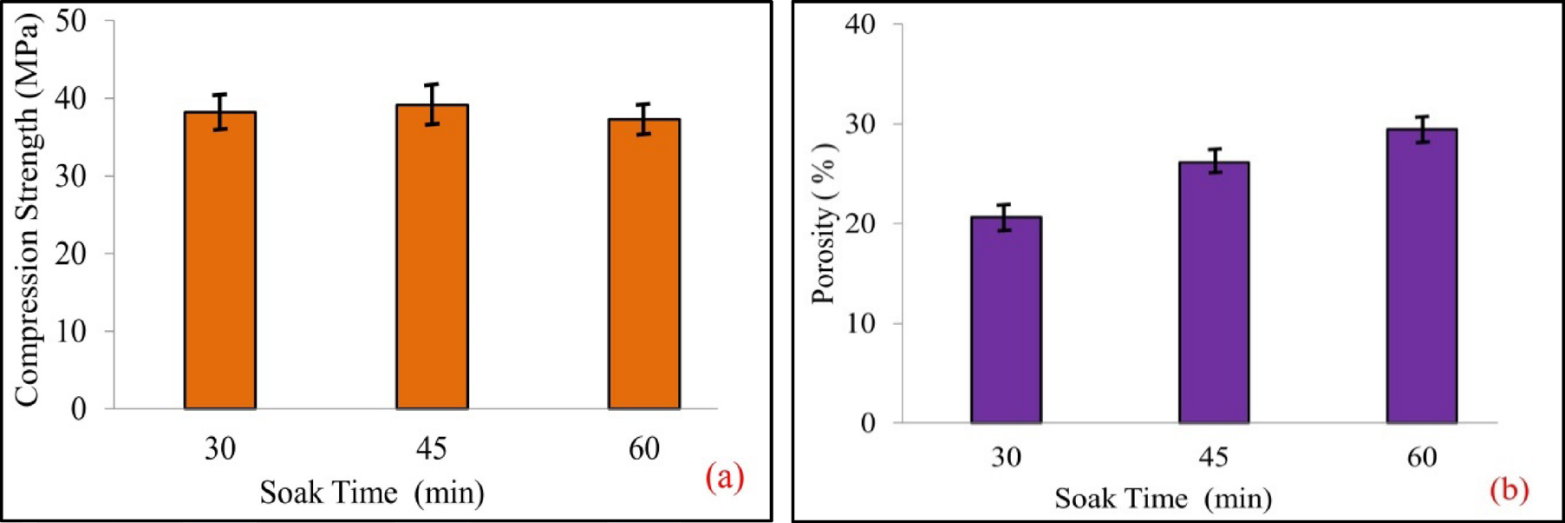
Influence of soak time on (**a**) compressive strength, (**b**) porosity.

Figure 7a shows impact of soak duration on the compressive strength of the sintered pellet at the ramp temperature rate of 35 °C/min. It is observed that compression strength gradually rises up to a soak time of 45 min and then gradually decreases over time for a soak time of 60 min. Figure 7b shows the effect of soak time duration on the porosity of sintered pellet, the porosity increases progressively as the soak time increases. The porosity is minimum as 20.658% when the soak time is 30 min and reaches the maximum value of 29.458% when the soak time is 60 min. A longer soak time for sintering a pellet, exceeding 30 min, will result in abnormal grain growth, α-TCP phase formation, and ultimately, reduced material properties after experimentation.

**Fig. 8:**
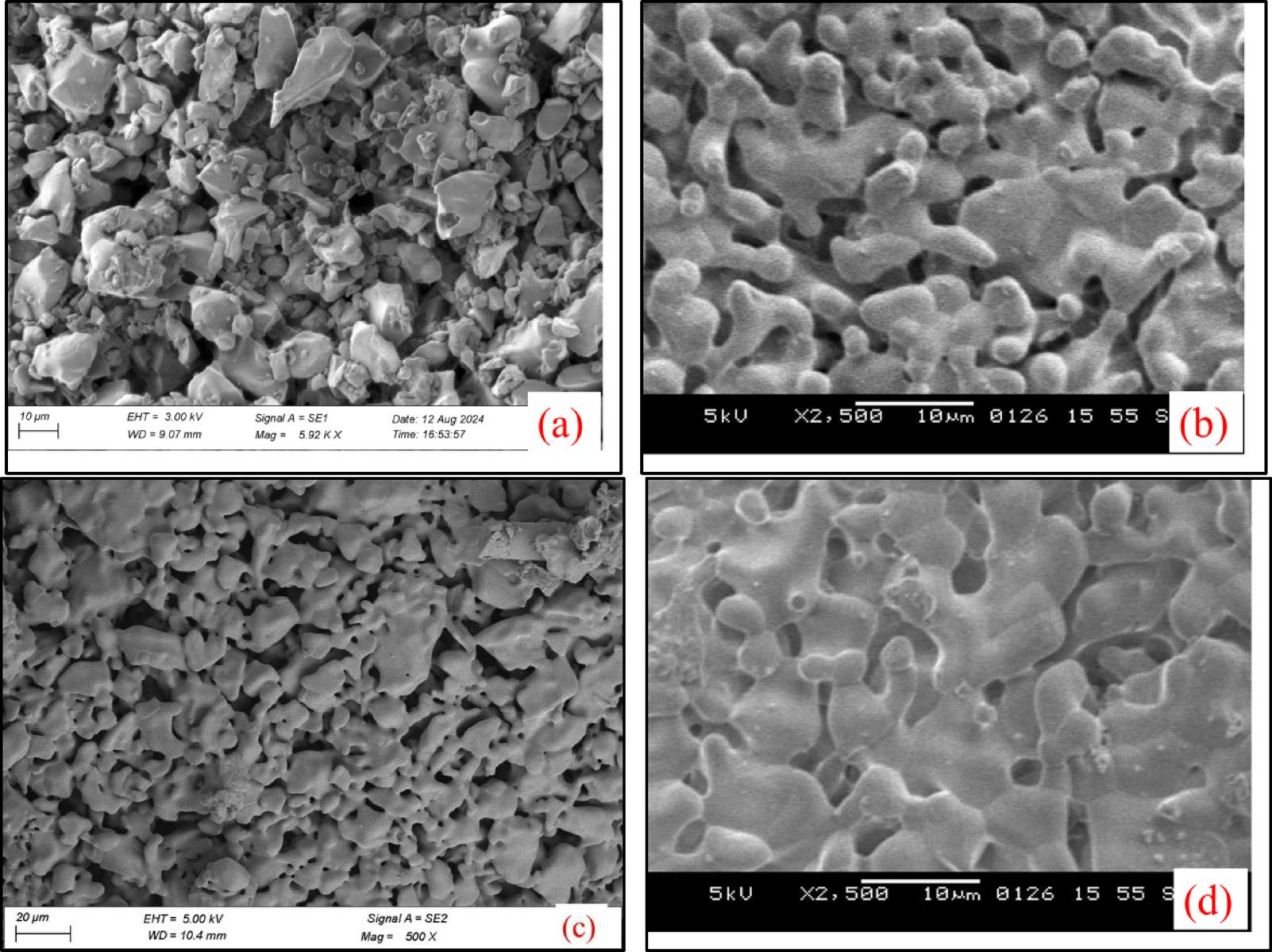
SEM micrographs of sintered pellet for different soak time durations indicates the phase transformation at (**a**) 0 min, (**b**) 30 min, (**c**) 45 min, (**d**) 60 min.

**Fig. 9:**
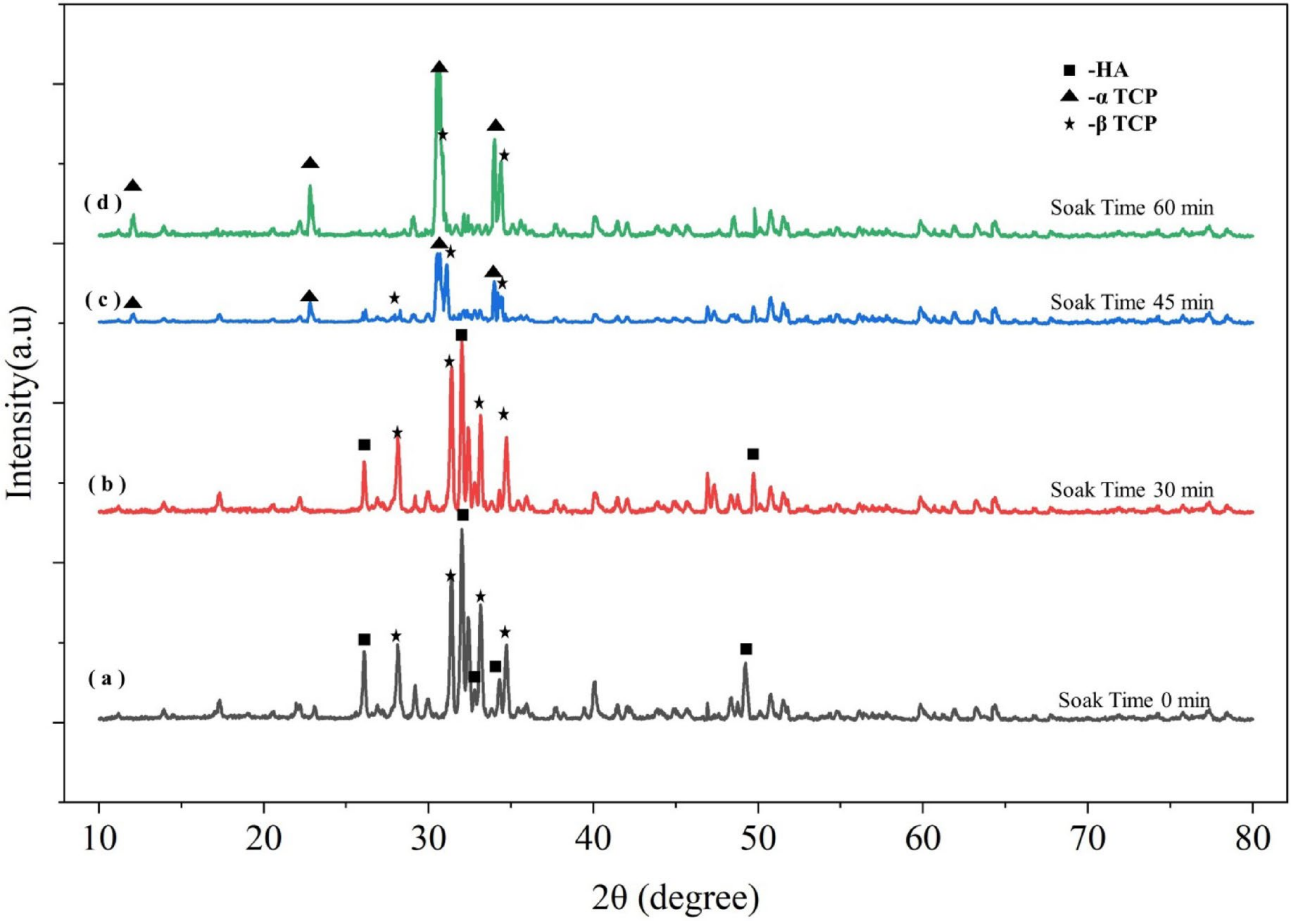
XRD plot for sintered pellet confirms the phase transformation with different soak time.

Figures 8 and 9 shows SEM micrographs and XRD plot results of the sintered pellet for different soak time duration respectively. Figure 8a shows that powder particles are very much in the β-TCP state at a soak time of 0 min. As soak time duration increases β-TCP transform into α-TCP phase as shown in Fig. 8c. Figure 9a shows the broad diffraction peaks represents biphasic structure having mixture of HA and TCP. Figure 9b shows that peaks are sharpened significantly compared to (a), showing increased crystallinity, and represents the β-TCP structure in major phases with some HA. Figure 9c shows α-TCP as major phase and β-TCP as weak phase structure. Figure 9d shows the sharpest peaks, represents full transformation into α-TCP phase structure and shows well-crystallized phase. As the soak time interval further increases, the liquid phase is connected to one piece, causing some TCP grains to fall off with the dissolution of the liquid phase, which creates holes or pits once it cools down.

Table 2 lists the porosity and mechanical performances of other HA-TCP ceramic pellets with different sintering conditions. It has been observed that the results obtained vary with different sintering conditions. The results obtained are recorded in Table 2, which shows that the porosity and compressive strength of microwave-sintered HA-TCP pellet ceramics have undergone significant changes. It can be noted that the manufacturing of high-performance bone scaffolds of HA-TCP ceramic materials is of great significance with the rapid development of microwave sintering technology. Shrinkages of 6–7% were observed during the study analysis.

**Table 2:** Effect of ramp temperature rate and soak time on mechanical property of sintered pellet.

Sr. No.	Sintering Temp. (ºC)	Ramp temperature rate (ºC/min)	Soak time (min)	Porosity (%)	Compressive strength
1	1200	15	30	22.095	37.33
2	1200	25	30	28.450	34.157
3	1200	35	30	20.658	38.21
4	1200	35	45	26.158	39.15
5	1200	35	60	29.458	37.33

Previous studies have reported a broad range of mechanical responses for porous bone-like materials. Wahab^47^ achieved 20% porosity after sintering. Morgan^48^ reported compressive strengths between 1 and 30 MPa with porosities of 30–90%, while Oftadeh^49^ reported strengths of 2–45 MPa within the same porosity range. Sun^50^ documented a compressive strength of 30 MPa at 40% porosity, whereas Metsger^51^ and Abbas^52^ reported strengths of 9.3 MPa and 15.6 MPa, respectively, for scaffolds with 50% porosity. Collectively, these findings highlight that the mechanical performance achieved in the present study is at the higher end of the reported range, underscoring the effectiveness of the optimized scaffold architecture.

Ceramic scaffold exhibits compressive strength of 2-65 MPa, whereas metallic scaffold have 45-100MPa^53,54^. The optimized scaffold exhibits a compressive strength of 39.15 MPa at 29.458% porosity, offering a rare combination of mechanical robustness and controlled porosity that effectively bridges the properties of cortical and cancellous bone. In contrast to metallic implants such as titanium or stainless steel which introduce severe mechanical mismatch leading to stress shielding, bone resorption, and eventual implant loosening, the present ceramic–polymer composite closely aligns with the natural mechanical environment of cancellous bone (2–45 MPa, 30–90% porosity). This biomechanical compatibility provides adequate support for load-bearing defects while minimizing the risk of revision surgery by enabling optimal stress transfer and physiological load sharing, thereby promoting faster and more natural bone regeneration.

## Conclusions

The highlight of this study is the integration of microwave sintering technology with a systematic parametric study for fabricating optimized biphasic HA-TCP bioceramics with controlled porosity and mechanical properties, which supports the development of advanced bone scaffolds. The microwave sintering route uniquely enables rapid, energy-efficient fabrication of bioceramics with desirable microstructural features critical for biomedical applications.

In the present study, sintering of HA-TCP ceramic pellets using microwave energy was investigated. The study refers to the impact of ramp temperature rate and soak time on the porosity and compressive strength on a pellet formed with 20% binder having 3% PVA concentration. The major findings of this study are as follows:

- Porosity of 20.658% and compressive strength of 38.21 MPa is recorded for the ramp temperature rate of 35 °C/min and soak time of 30 min.
- During sintering process, certain ramp temperature rate around 35 °C/min, is necessary for the densification. Porosity of the pellet increases from 22.095% to 28.450% when ramp temperature rate increases from 15 to 25 °C/min.
- For a ramp temperature rate of 35 °C/min, soak time increases from 30 min to 45 min; porosity also increases to 26.158%. At 1200 °C, this prolonged soak time allows for the formation of α-TCP from β-TCP, and the compressive strength increases to 39.15 MPa.
- As soak time increases to 60 min, porosity increases to 29.458%, but holding for this long time allows conversion of α-TCP phase to β-TCP phase, resulting in a decrease in compressive strength to 37.33 MPa.
- To achieve optimum results for a bone scaffold made up of HA-TCP ceramic using microwave sintering, a ramp temperature rate of 35 °C/min and a soak time of 45 min give adequate results.

## Data Availability

The datasets used and/or analyzed during the current study are available from the corresponding author (Bhupesh Sarode) on reasonable request.
